# Real-Time Monitoring of Grazing Cattle Using LORA-WAN Sensors to Improve Precision in Detecting Animal Welfare Implications via Daily Distance Walked Metrics

**DOI:** 10.3390/ani13162641

**Published:** 2023-08-16

**Authors:** Shelemia Nyamuryekung’e, Glenn Duff, Santiago Utsumi, Richard Estell, Matthew M. McIntosh, Micah Funk, Andrew Cox, Huiping Cao, Sheri Spiegal, Andres Perea, Andres F. Cibils

**Affiliations:** 1 Division of Food Production and Society, Norwegian Institute of Bioeconomy Research (NIBIO), PB 115, N-1431 Ås, Norway; 2 Department of Animal and Range Sciences, New Mexico State University, Las Cruces, NM 88003, USA; glennd@nmsu.edu (G.D.); funkm@nmsu.edu (M.F.); arcox@nmsu.edu (A.C.); arperea@nmsu.edu (A.P.); 3 United States Department of Agriculture-Agriculture Research Service, Jornada Experimental Range, Las Cruces, NM 88003, USA; rick.estell@usda.gov (R.E.); mattmac@nmsu.edu (M.M.M.); sheri.spiegal@usda.gov (S.S.); 4 Department of Computer Science, New Mexico State University, Las Cruces, NM 88003, USA; hcao@nmsu.edu; 5 United States Department of Agriculture Southern Plains Climate Hub, United States Department of Aagricultulre-Agriculture Rearch Services, Oklahoma and Central Plains Agricultural Research Center, El Reno, OK 73036, USA; andres.cibils@usda.gov

**Keywords:** precision livestock farming, precision livestock ranching, internet of things, long range wide area network

## Abstract

**Simple Summary:**

Global positioning system (GPS) coordinates are often used to calculate distance traveled, a useful metric for research and decision-making processes in livestock management. The study aimed to determine the accuracy of using LoRa-WAN sensors to measure the walking distances of grazing cattle in real time. The study compared the accuracy of distance computation using real-time LoRa-WAN sensed GPS alone or in combination with motion data from triaxial accelerometers. The analysis showed that the fusion of GPS and accelerometer data was more suitable for calculating walking distance in detecting animal welfare implications associated with immobility.

**Abstract:**

Animal welfare monitoring relies on sensor accuracy for detecting changes in animal well-being. We compared the distance calculations based on global positioning system (GPS) data alone or combined with motion data from triaxial accelerometers. The assessment involved static trackers placed outdoors or indoors vs. trackers mounted on cows grazing on pasture. Trackers communicated motion data at 1 min intervals and GPS positions at 15 min intervals for seven days. Daily distance walked was determined using the following: (1) raw GPS data (RawDist), (2) data with erroneous GPS locations removed (CorrectedDist), or (3) data with erroneous GPS locations removed, combined with the exclusion of GPS data associated with no motion reading (CorrectedDist_Act). Distances were analyzed via one-way ANOVA to compare the effects of tracker placement (Indoor, Outdoor, or Animal). No difference was detected between the tracker placement for RawDist. The computation of CorrectedDist differed between the tracker placements. However, due to the random error of GPS measurements, CorrectedDist for Indoor static trackers differed from zero. The walking distance calculated by CorrectedDist_Act differed between the tracker placements, with distances for static trackers not differing from zero. The fusion of GPS and accelerometer data better detected animal welfare implications related to immobility in grazing cattle.

## 1. Introduction

Store-on-board telemetry devices, including Global Positioning System (GPS) loggers and accelerometer sensors, have gained traction in the last two decades for studying livestock and wildlife grazing behavior. Integrating these unobtrusive telemetry devices has enabled the scientific community to gain insights into the behaviors of different animals in their natural settings [1,2,3,4]. For instance, studies performed on cattle grazing on extensive rangelands using GPS loggers have documented animal movement in correlation with foraging activities influenced by resource distribution, in their search for thermal comfort, to avoid predator presence in their environment or other essential activities [3,5,6,7]. Hence, the mobility of animals within their environment is crucial for their survival, as they respond to various biotic and abiotic stimuli.

Developments in communication technology have facilitated real-time data transmission from telemetry devices, helping advance Precision Livestock Farming/Ranching (PLF/R) applications. One such technology is the Long Range Wide Area Network (LoRa WAN), a wireless low-power data transmission system with bidirectional capabilities, enabling data packet transmission and configuration commands to be sent remotely [8,9,10,11]. These networks exhibit far-reaching coverage (up to 10 km) with a stronger signal strength compared to Wi-Fi and Bluetooth, penetrating insulated objects, and requiring minimal maintenance with a longer lifespan [11,12]. As a result, LoRa WAN offers an affordable and effective solution for implementing PLF/R on extensive rangelands with limited connectivity [11,12,13].

The Internet of Things (IoT) is at the heart of these applications, with sensory devices that process unique measurements of physiological and behavioral parameters on animals, gateways that enable data transmissions from sensors to the internet, cloud services that provide data storage and analysis, with an output application layering tailoring the data into usable information for a particular end-user [10,14,15,16].

Integrating PLF/R could facilitate the transition of traditional livestock production systems that place emphasis on maximizing animal output to aspirational management systems that optimize production efficiency by increasing animal welfare and whole-farm sustainability [17,18,19]. In addition, consumer pressure exerted through purchasing power in most developed countries has demanded livestock producers increase animal welfare transparency via enhanced traceability [16,20,21]. Also, the continuous monitoring application of the PLF/R technology in animal production would enable real-time management of the smallest manageable unit (sensor-based animal-specific attention) on the production front, enhancing management flexibility and minimizing environmental impacts with alleviation of intensive labor requirements [20,22,23].

Animal welfare is a multidimensional concept related to the repertoire of behaviors performed by an animal in its natural state that promote ‘normal’ biological functions [20,24]. Therefore, the success of the PLF/R application to address animal welfare will depend on the accuracy of sensors to detect changes associated with a deteriorating animal state [5,18,25]. For instance, using frequently acquired GPS data to compute daily distance traveled can have direct management implications in addressing animal welfare on extensive cattle production [17,26,27]. However, control of data quality and improvements in analytical procedures (algorithms for calculating daily distance traveled metrics) are paramount in facilitating the implementation of PLF/R applications to address issues stated previously.

For instance, the precision of GPS measurements is affected by several factors, including the type of device with internal limitations, the sensor environment placement, and the timing of data acquisition in relation to the orbiting satellites. Precision ranges for GPS sensors used in animal tracking have been reported to not exceed 30 m, provided that the units have a relatively unobstructed view of the sky [28,29]. However, the precision error varies among devices and can be further affected by obstructions in the communication pathways with orbiting satellites [29,30,31,32]. Therefore, flagging erroneous GPS locations is vital when incorporating the data into a computation informing a PLF/R application.

This study aimed to assess the effectiveness of real-time sensors using Long Range Wide Area Network (LoRa-WAN) communication for monitoring animal welfare through daily distance travel metrics. To achieve this, we evaluated the accuracy of calculating the daily distance travel metric using global position system (GPS) data alone or in combination with motion data derived from triaxial accelerometers. Three algorithms were tested to detect any differences between the static sensors placed outdoors (Outdoor, n = 6) or indoors with an obstructed view of satellites (Indoor, n = 5), vs. trackers mounted on grazing cows (Animal, n = 6). We hypothesized that the daily distance traveled by the animal tracker would be greater than the static trackers, with no significant difference between outdoor and indoor means. We also predicted that the daily distance traveled by the static trackers would be negligible and not differ from zero, as an animal welfare alert system would require immobility detection.

## 2. Materials and Methods

### 2.1. Experimental Site and LoRa Trackers Configuration

**Figure 1:**
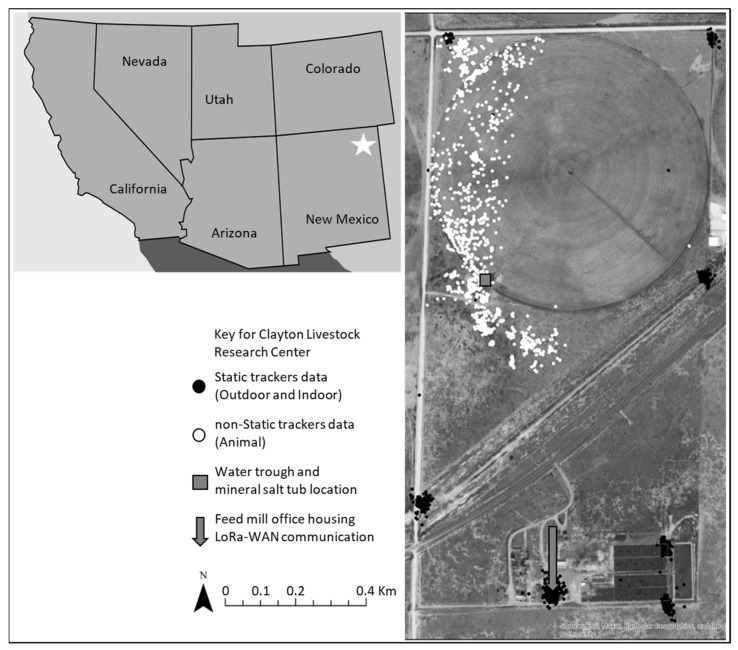
Top left: Reference map of the Southwest United States showing the research site marked with a star (Clayton, New Mexico). Right: Map of New Mexico State University’s Clayton Livestock Research Center (CLRC) displaying GPS locations of static trackers and non-static trackers, along with other important landmarks on the research site.

The study was conducted at the New Mexico State University’s Clayton Livestock Research Center (NMSU CLRC), which is located 7 miles east of Clayton, New Mexico, USA, and covers a total area of 1.39 km^2^ (320 acres). The research site consists of flat terrain, with a section of 0.79 km^2^ (195 acres) of fenced land configured with a center pivot winter wheat (*Triticum aestivum* L.) irrigated pasture and a feedlot facility housed with hydraulic chutes for animal handling south of the section (Figure 1).

Long Range Wide Area Network (LoRa WAN) was chosen as the mode of communication between an antenna and the trackers (widgets) [8,9,15]. The PLF/R system at the NMSU (CLRC) consisted of a single Kerlink^®^ LoRa-WAN 915 MHz gateway (https://www.kerlink.com/, accessed on 15 October 2020) with an external high gain antenna mounted on the feed mill tower using a 30 m coaxial cable (Figure 1). The gateway was purchased with a licensed software platform (Thingpark) developed by Actility^®^ (https://www.actility.com/, accessed on 15 October 2020), allowing data traffic monitoring and gateway functionality.

LoRa WAN-enabled Abeeway^®^ (https://www.abeeway.com/, accessed on 10 October 2020) Industrial Trackers US915 were configured to communicate with a single gateway at the NMSU (CLRC) site. The trackers weigh 240 g, are housed in a waterproof casing, and operate using an internal Lithium-thionyl Chloride Type D battery (14 Ah/3.6 V). The trackers were equipped with position, motion, and temperature sensors, and a LoRa-WAN communication chip embedded in their motherboard. The trackers were then contained within a Pelican^®^ R20 Ruck case strapped on an adjustable nylon collar belt to reinforce structural integrity and waterproofing capabilities [11]. 

The industrial trackers had a licensed software platform (Abeeway Device Manager 2.13.0) for the data surveillance (Map, Performance Monitor, and Uplinks data log) and tracker configuration under an annual subscription [11]. We opted for the “Activity tracking” configuration, with activity reporting as the “main operational” mode and periodic position message as a “side operation.” The data collection interval was set at 1 min for motion detection using the tri-axial accelerometer sensors and 15 min intervals for position acquisition using the GPS-only option.

The process of GPS acquisition demands significant power, leading to a dilemma between prioritizing high-frequency, short-duration experiments, or low-frequency data collection for longer-duration studies [4]. According to the guidelines for LoRa WAN-enabled Abeeway^®^ Industrial Tracker, the estimated device duration is 20 months when acquiring 24 positions per day (at an hourly frequency of GPS data collection). The selected motion and position acquisition scheduling for the study was hypothesized to provide a battery duration of approximately 5 months, making these trackers suitable for deployment on a working ranch with minimal livestock interaction. 

### 2.2. Study Deployment, Animals

From 24 October to 17 November 2020, 6 randomly selected trackers (Outdoor) were positioned in fixed locations at incremental distances from the LoRa WAN gateway (Figure 1). Using adjustable belts, the trackers were secured on an existing fence line, approximately ~1 m above the ground and facing the antenna. In addition, five trackers were housed inside the feed mill office (Indoor), adjacent to the LoRa WAN gateway, to simulate obstructed GPS communication with orbiting satellites (Figure 1). The deployment lasted 24 days, but no data was collected for 15 days due to gateway software maintenance during the middle of deployment (27 October to 11 November 2020).

A follow-up deployment from 22 December to 31 December 2020, utilized the trackers mounted on mature cattle (Animal) with a subset of only six trackers out of the eleven randomly selected for the static phase utilized for analysis. Trackers were safely secured on the necks of the animals using adjustable nylon belts. The collared animals grazed on native grasslands with access to a portion of an irrigated winter wheat pasture, with ad libitum access to water and a mineral salt tub (Figure 1). Animal use was approved by the New Mexico State University Institutional Animal Care and Use Committee (protocol # 2019-008).

### 2.3. Data Processing

All the trackers’ payloads were routed from the Thingpark server that populated the Abeeway Device Manager to a local New Mexico State University server for data retrieval and analysis. The data from the static phase were trimmed, with the initiation and termination dates excluded from the analysis. In addition, the dates when the gateway was under maintenance (27 October to 11 November 2020) were also excluded. A total of 7 days of deployment from the static trackers (Indoor, n = 5 and Outdoor, n = 6) were analyzed. In the follow-up phase with the trackers mounted on mature cattle (Animal, n = 6), animals were allowed three days to acclimate to collars and the new environment, followed by a 7-day data collection period (25 December to 31 December 2020). 

**Figure 2:**
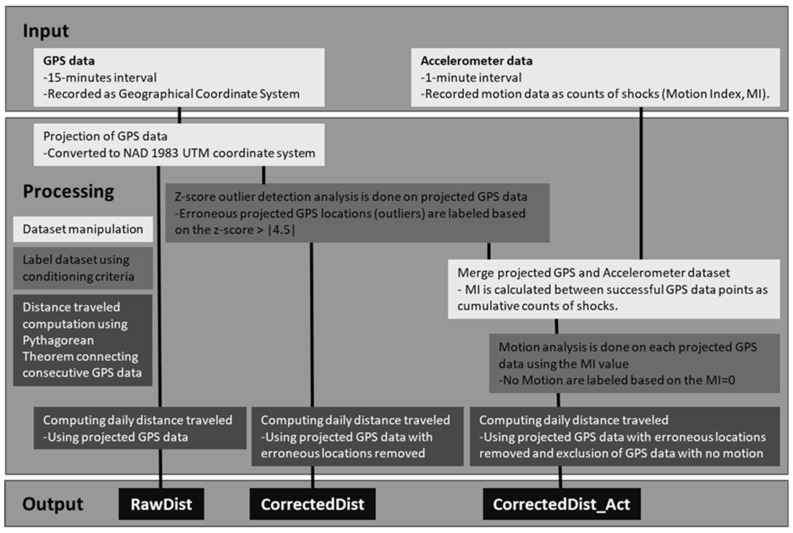
Data flow diagram illustrating input data from the LoRaWAN trackers and various dataset processing steps prior to calculating daily distance traveled using three algorithms: RawDist, CorrectedDist, and CorrectedDist_Act.

GPS coordinates were projected to NAD 1983 UTM coordinate system (Zone 13 N) using ArcGIS software (ESRI 2018, ArcMap Desktop v. 10.6). Erroneous GPS locations were filtered using a z-score outlier detection analysis for the northing and easting coordinates separately, as described by Nyamuryekung’e et al. [33]. The z-score outlier detection analysis followed the conversion of daily projected coordinate values for an individual tracker into a normalized z-score, highlighting extreme score values with low probability under assumptions for a normal distribution of data points (z > |4.5|) [33]. Motion data were reported as counts (Motion Index, MI) of shock within the interval of data acquisition across the triaxial accelerometer using internal default threshold values and were represented as cumulative counts between successful GPS data (Figure 2).

The three algorithms for daily distance traveled included using (1) raw GPS data with associated erroneous locations (RawDist), (2) GPS data with erroneous locations removed using z-score > |4.5| analysis (CorrectedDist), or (3) GPS data with erroneous locations removed combined with the exclusion of GPS data associated with no motion (MI = 0) from the triaxial accelerometer reading (CorrectedDist_Act) (Figure 2). Using projected GPS positions for the Static (Indoor and Outdoor) and non-Static (Animal) trackers, we calculated daily distances traveled (m) using three algorithms by summing the consecutive GPS distances calculated using the Pythagorean Theorem within a day, as described by Nyamuryekung’e et al. [34]. 

The daily distance traveled calculations followed the steps below with their mathematical equations:

1. Let consecutive GPS position be represented as (*x*_1_, *y*_1_), (*x*_2_, *y*_2_), (*x*_3_, *y*_3_), …, (*x_n_*, *y_n_*) where n is the total number of daily GPS positions.
2. Pythagorean Theorem for calculating the distance between two consecutive GPS positions (*x_i_*, *y_i_*) and (*x*_(*i*+1)_, *y*_(*i*+1)_):

$$Distance=\left(\sqrt{{\left(x_{i}-x_{\left(i+1\right)}\right)}^{2}+{\left(y_{i}-y_{\left(i+1\right)}\right)}^{2}}\right)$$

3. The daily distance traveled within a day was calculated by summing the distance between all consecutive GPS positions within a day:

$$\begin{array}{l} Daily\;distance=\left(\sqrt{{\left(x_{1}-x_{2}\right)}^{2}+{\left(y_{1}-y_{2}\right)}^{2}}\right)+\left(\sqrt{{\left(x_{2}-x_{3}\right)}^{2}+{\left(y_{2}-y_{3}\right)}^{2}}\right)+\\ \ldots +\left(\sqrt{{\left(x_{\left(n-1\right)}-x_{n}\right)}^{2}+{\left(y_{\left(n-1\right)}-y_{n}\right)}^{2}}\right) \end{array}$$

Caution GPS coordinates must be projected to provide the daily distance traveled by the tracker in meters.

### 2.4. Data Analysis

An analysis based on descriptive statistics on each daily distance measurement (RawDist, CorrectedDist, and CorrectedDist_Act) was computed using the MEANS procedure in SAS 9.3 (SAS Institute, Cary, NC, USA). The data were grouped according to tracker placement (Indoor, Outdoor, or Animal) and the calendar date of the deployment. Mean, standard error, sample size, and minimum and maximum values for each daily distance measurement (RawDist, CorrectedDist, and CorrectedDist_Act) were computed for each unique combination of categorical grouping (Placement × Date).

The daily distance measurements (RawDist, CorrectedDist, and CorrectedDist_Act) were estimated using SAS 9.3 (SAS Institute, Cary, NC, USA). The MIXED procedure with a ‘covtest’ statement was used to analyze distances via one-way ANOVA comparing treatments of trackers’ placement either inside a building (Indoor), on the field (Outdoor), or mounted on mature cows (Animal) grazing pastures at the research site (Ho: µIndoor = µOutdoor = µAnimal). The tracker’s ID (n = 11), a categorical classification for each tracker ID and placement combination (n = 17), in addition to the dates (n = 14) of deployment, were modeled as random variables. Means were computed and compared via LSMEANS statement for each daily distance measurement (RawDist, CorrectedDist, and CorrectedDist_Act) between the treatments of tracker placement (Indoor, Outdoor, or Animal), with a ‘pdiff’ statement for pairwise comparison. In addition, t-tests were conducted within each model to determine if the daily distance metrics (RawDist, CorrectedDist, and CorrectedDist_Act) calculated for each tracker placement (Indoor, Outdoor, or Animal) differed from zero (Ho: µ = 0). Lastly, using the estimate statement, a comparison of the means between the static state (Indoor and Outdoor) and non-static trackers (Animal) was computed (Ho: µStatic = µnon-Static). For all procedures, differences were declared statistically detectable at *p* ≤ 0.05.

## 3. Results

**Figure 3:**
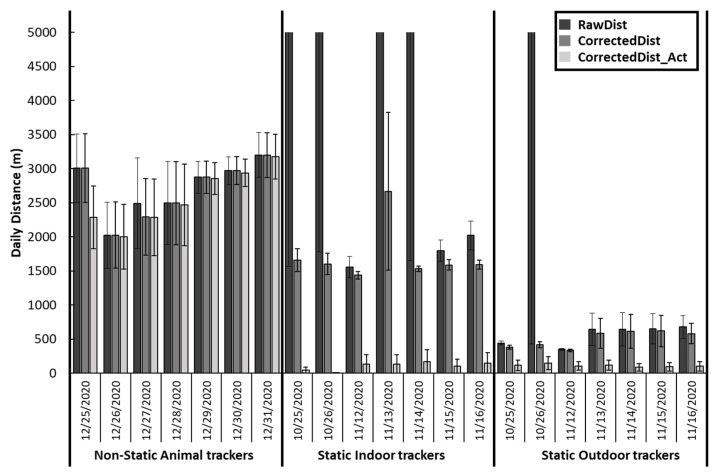
Mean daily distance with standard error bars calculated using three algorithms (RawDist, CorrectedDist, and CorrectedDist_Act), categorized by tracker placement (Animal, Indoor, or Outdoor) and the calendar date of deployment.

The descriptive analysis revealed variability in the accuracy of each daily distance measurement (RawDist, CorrectedDist, and CorrectedDist_Act) in combination with the treatment of the tracker’s placement (Indoor, Outdoor, or Animal). Overall, the RawDist measurement computed using the indoor trackers had the highest means and standard error. In contrast, the daily distance calculated using CorrectedDist_Act for the outdoor trackers had the lowest means and standard error. However, animal trackers exhibited the least variability in the means when compared between the three daily distance measurements (RawDist, CorrectedDist, and CorrectedDist_Act) (Figure 3).

The results from our analysis indicated no significant relationship between RawDist measurement and the treatment of tracker placement (*p* = 0.23) when the assumption of normal distribution was violated (outliers detected) (Table 1). Due to the low precision, the means of the treatments (Indoor, Outdoor, or Animal) were not different from zero. In addition, there was no effect on the estimate comparison between static and non-static groupings of the means.

The CorrectedDist measurement revealed a significance of treatment (*p* < 0.01), with animal trackers covering a greater daily distance than either indoor or outdoor placement (Table 1). However, indoor and outdoor means were different from each other (*p* < 0.01). In addition, indoor and animal placement trackers differed statistically from zero (Ho: µ = 0; *p*_Indoor_ < 0.01, *p*_Outdoor_ = 0.06, *p*_Animal_ < 0.01). However, the estimate statement indicated a significant difference (*p* < 0.01) between the static and non-static groupings of the means.

With the last method of calculating the distance, the CorrectedDist_Act measurement was also affected by tracker placement (*p* < 0.01). Animal trackers covered a greater daily distance compared to either indoor or outdoor placement. In addition, indoor and outdoor means were not different from each other (*p* < 0.62). Only the animal trackers were significantly different from zero (Ho µ = 0; *p*_Indoor_ = 0.84; *p*_Outdoor_ = 0.38; *p*_Animal_ < 0.01) (Table 1). The estimate statement also indicated a significant difference (*p* < 0.01) in the means grouping of Static vs. non-Static trackers.

## 4. Discussion

To achieve user-friendly PLF/R systems, data flow must be near real time for a farmer or rancher to monitor an individual animal’s health, welfare, and yields [17,20]. However, it is worth noting that the development of the PLF/R platform depends on the advancement of precision and accuracy within sensors used in the IoT ecosystem. This is because the PLF/R system is data-driven, emphasizing the need for high-quality data [35]. While the future of PLF/R lies in data-driven techniques like machine learning (ML) and deep learning (DL) to identify data patterns, it is equally important not to overlook the value of mechanistic modeling approaches based on the conceptual understanding of system dynamics (hypothesis-centered) [18,36]. While robust data-driven approaches (ML and DL) often lack transparency in their predictions, mechanistic models use animal performance parameters for prediction. Therefore, a hybridized approach that integrates both data-driven and mechanistic modeling methodologies is warranted for enhanced PLF/R outcomes [18,36].

The distance traveled is a standard metric calculated from GPS coordinates with significant applications in research and management decision-making processes [3,4,26]. The objectives of this study were to test the reliability of the daily distance traveled metric calculation using three algorithms (GPS data alone or in combination with motion data) for detecting the differences between static trackers placed either outdoors or indoors with an obstructed view of orbiting satellites vs. trackers mounted on cows grazing on pasture. We hypothesized that the daily distance traveled metrics would be higher for the animal-mounted trackers than the static trackers and that there would be no difference between the two types of static trackers (Outdoor = Indoor), and that the daily distance traveled by the static trackers (Outdoor or Indoor) would not differ from zero.

### 4.1. GPS Accuracy Measurement on Static Trackers

An analysis of GPS data accuracy in this study showed that there were infrequent erroneous GPS locations that worsened the distance computational means output (RawDist) when they were integrated into the model. A related study that utilized GPS data from static indoor and outdoor trackers found that outdoor trackers had greater accuracy in remote sensing of GPS locations. This is because indoor trackers experience increased communication interference in acquiring satellite signals. The study’s findings indicated that 95% of GPS data points fell within a radius of 15 and 40 m for outdoor and indoor trackers, respectively [33]. The study concluded that these trackers showed comparable accuracy to other devices available in the market, with a position bias calculated by excluding erroneous GPS positions using the Euclidean distance between the tracker’s actual location (Stationary) and the projected GPS points averaging 5.20 and 17.76 m for outdoor and indoor trackers, respectively [33].

In pasturelands with a canopy cover, obstructed GPS data acquisition is common. For instance, previous studies collaring goats herded on rugged terrain, and those that calculated horizontal accuracy between trackers placed in the open field and under canopy cover both reported low accuracy for trackers with obstructed views of orbiting satellites [29,31]. Therefore, it is essential to include a pre-processing phase to detect outliers in GPS data before any further analysis [33,37].

### 4.2. Daily Distance Traveled Calculated Using RawDist

The computation of distance traveled using raw GPS data (RawDist) did not support our first hypothesis, as there was no detectable difference between the tracker placements. Additionally, statistical model assumptions associated with outliers were violated, so caution is necessary when interpreting these results. This underscores the need for internal algorithms to filter out extreme GPS outliers. RawDist had the highest numerical value compared to the other two computations (CorrectedDist and CorrectedDist_Act) used to calculate the distance traveled. McGavin et al. [27] also found that including GPS data with low accuracy increased the calculated distance. Furthermore, the RawDist measurement failed to support our second hypothesis, as the indoor trackers differed from zero, and there was no difference in the comparison between the static and non-static trackers. This failure of RawDist measurement suggests that this algorithm has low accuracy in detecting animal welfare implications associated with immobility.

### 4.3. Daily Distance Traveled Calculated Using CorrectedDist

The corresponding analysis for screening erroneous GPS positions using the z-score, as proposed by Nyamuryekung’e et al. [33], improved the normal distribution of the daily distance metric (CorrectedDist), leading to partial support for our first hypothesis, which predicted that the Animal tracker would cover a greater daily distance than the indoor and outdoor trackers. However, indoor and outdoor trackers differed from each other. CorrectedDist also failed our second hypothesis, as the distance means of the static trackers differed from zero. Due to the random errors in GPS positioning equipment, the static trackers registered a significant daily distance measurement that was different from zero, making the analysis sensitive to inflated error distance measurements in situations with a high temporal frequency of GPS acquisition and low GPS location accuracy. Ganskopp and Johnson [32] and McGavin et al. [27] also found a correlation between short GPS sampling intervals and overestimated distance calculation. However, CorrectedDist was able to detect differences between static and non-static trackers. Therefore, CorrectedDist has some utility for use in animal welfare metrics to detect differences between static and non-static states, but it might fail to statistically detect a zero daily distance measurement on static trackers.

### 4.4. Daily Distance Traveled Calculated Using CorrectedDist_Act

The final model, which excluded GPS data with erroneous locations and GPS data associated with no motion from the accelerometer reading (CorrectedDist_Act), supported our first hypothesis, showing that the Animal tracker covered a greater daily distance than both indoor and outdoor trackers, which did not differ from each other. Moreover, CorrectedDist_Act also supported our second hypothesis, with the daily distance covered by static trackers not differing from zero. As the static trackers were mostly stationary, activity data was almost non-existent, with only three trackers contributing to the daily distance (Indoor n = 1 and Outdoor n = 2). CorrectedDist_Act also detected the difference between static and non-static trackers. Therefore, CorrectedDist_Act is the recommended metric for daily distance measurement, with acceptable accuracy in detecting animal welfare implications associated with immobility.

### 4.5. Limitations of the Daily Distance Traveled Calculations

The selection of the GPS frequency acquisition has significant implications for calculating the distance traveled by animals. A high GPS frequency acquisition can lead to an overestimation of the distance traveled due to the inclusion of GPS positions with inherent position bias, as well as increased battery drainage of the trackers [4,32]. On the other hand, low GPS frequency acquisition may result in underestimating the distance traveled, as it may miss the sinuosity of the actual path taken by the animals [38]. Achieving a balance in selecting the GPS frequency acquisition is crucial to avoid both overestimation and underestimation of the true distance, and it is essential to consider the spatial extent to which the animals are operating. The chosen interval for GPS frequency acquisition in this study is believed to adequately represent the distance traveled by animals in extension operations [3].

Improvements in CorrectedDist_Act can be achieved by calibrating the triaxial accelerometer data to represent the animal’s time budget, thus improving the accuracy of distance traveled estimation from GPS data by excluding a resting activity from the analysis [32]. Studies have shown that including resting and inactive GPS positions when calculating the distance traveled can artificially increase it by approximately 15.2% [32]. However, it is important to note that the purpose of this experiment did not involve time-budget calibration of the accelerometer data. The 1 min interval motion intensity measurements in this study revealed clear diurnal patterns consistent with grazing animals’ behavior [33]. For example, intense grazing events are typically observed around dusk, which was evident from the accelerometer data with a peak of intensity around 1800 h [33]. Future analysis will explore decoding the activity messages into time budgets for the animals [39,40]. Additionally, accurate classification of the activity data can further enhance the filtering of erroneous GPS data, as suggested by Muminov et al. [37], who used maximum animal movement likelihood criteria based on activity classification to filter out erroneous GPS data.

## 5. Conclusions

The user interface or dashboard application is arguably the most valuable component in the Internet of Things (IoT) ecosystem, particularly in a Precision Livestock Farming and Ranching (PLF/R) platform. This is because the data collected by the sensory devices are transformed into information tailored to a specific end-user through the user interface platform. In a PLF/R system, the dashboard application assists ranchers in decision-making processes. Hence, the metrics presented in the dashboard application must possess both high accuracy and precision. These findings emphasize the necessary sensitivity required to develop bio-sensing algorithms that can alert managers of animal welfare concerns. Similarly, the CorrectedDist_Act model emphasizes the importance of combining GPS and accelerometer data when calculating the walking distance of grazing cattle. Furthermore, the results highlight the value of integrating multiple sources of independent sensor data for an improved interpretation of data derived from PLF/R tools.

## Figures and Tables

**Table 1:** Least square means ± standard errors for a daily distance calculated using three algorithms: (RawDist, CorrectedDist, and CorrectedDist_Act). Statistical analysis involved comparing distance computations across tracker placements (letters) for non-static vs. static trackers (µ non-static = µ static), and assessing the differences of means from zero (µ = 0).

Distance (m)	State	Placement	LSmeans		*p*-Value	
			±Std Err		µ Non-Static = µ Static	µ = 0
RawDist *	non-Static	Animal	2724 ± 63,475	a	0.33	0.97
	Static	Indoor	150,644 ± 69,534	a		0.03
	Static	Outdoor	11,364 ± 66,543	a		0.86
CorrectedDist	non-Static	Animal	2695 ± 193	a	<0.01	<0.01
	Static	Indoor	1725 ± 211	b		<0.01
	Static	Outdoor	385 ± 202	c		0.06
CorrectedDist_Act	non-Static	Animal	2574 ± 186	a	<0.01	<0.01
	Static	Indoor	42 ± 203	b		0.84
	Static	Outdoor	170 ± 193	b		0.38

* Violation of statistical model assumptions associated with outlier presence.

## Data Availability

The raw data concerning the LoRa WAN sensors showcased in this study are available on request from the corresponding authors.
